# Racial and Geographic Disparities in Automated External Defibrillator Use During EMS Encounters in the United States

**DOI:** 10.3390/healthcare14101413

**Published:** 2026-05-21

**Authors:** Peter G. Kreysa

**Affiliations:** Department of Family and Consumer Sciences, California State University, Long Beach, CA 90840, USA; peter.kreysa@csulb.edu

**Keywords:** emergency medical services, EMS, automated external defibrillator, AED, health equity, racial disparities, geographic variation

## Abstract

**Highlights:**

**What are the main findings?**

- AED use during EMS encounters shows clear racial and geographic inequities, with White patients and suburban/rural/frontier regions experiencing the lowest deployment rates.
- Disparities persist even within clinically severe encounters such as cardiac arrest and high-acuity cases, indicating that clinical need alone does not explain variation in AED use.

**What are the implications of the main findings?**

- Improving AED placement, EMS deployment strategies, and community readiness, especially in underserved regions, may help reduce inequities in early defibrillation.
- Equity-focused EMS planning and targeted public-access defibrillation initiatives are needed to ensure that time-sensitive interventions are delivered consistently across diverse populations.

**Abstract:**

Background: Out-of-hospital cardiac arrest is a major cause of mortality, and survival depends heavily on rapid defibrillation. Automated external defibrillators (AEDs) can significantly improve outcomes when used before emergency medical services (EMS) arrive, yet access to and use of these devices remain uneven across communities. This study investigates racial and geographic disparities in AED use during EMS encounters in the United States, evaluating differences across racial groups, geographic settings, cardiac arrest status, and patient acuity, irrespective of whether a bystander or EMS personnel applied the device. Methods: This descriptive study used aggregated data from the National Emergency Medical Services Information System (NEMSIS) Public Release Data Cube to compare AED use across racial, geographic, cardiac arrest, and acuity categories. AED use was defined as any documented application during the EMS encounter. Results: The dataset included 106,246 EMS encounters across six racial and ethnic groups. AEDs were applied in 16,688 encounters (15.7%), with substantial variation across demographic and geographic categories. Asian, American Indian or Alaska Native, and Black or African American patients had the highest rates of AED use, while White patients had the lowest rate despite representing the largest share of encounters. Urban areas accounted for most AED deployments, whereas suburban and frontier regions showed markedly lower use, while rural AED use was similar to urban rates. AED application was strongly associated with cardiac arrest and high patient acuity, yet racial differences persisted even within these clinically severe categories. Conclusions: AED use generally aligns with clinical indicators such as cardiac arrest and critical acuity, but meaningful racial and geographic differences were observed, reflecting descriptive patterns rather than confirmed disparities. These patterns should be interpreted cautiously, as the aggregated nature of the dataset limits the ability to determine whether differences reflect inequities, incident characteristics, or EMS system factors. These findings highlight the need for targeted strategies to expand AED access, improve device placement, and strengthen community readiness in underserved areas. Integrating AED availability into broader EMS planning and community outreach may help reduce inequities and create conditions that support improved survival outcomes. Further research using individual-level data and geospatial methods is needed to clarify the drivers of these observed differences and inform equitable prehospital care policies.

## 1. Introduction

The global significance of equitable access to early defibrillation has been underscored by the Lancet Commission on Sudden Cardiac Death, which identifies timely defibrillation and robust public-access AED programs as essential components of a comprehensive strategy to reduce preventable mortality [1,2]. The Commission emphasizes that disparities in AED availability, community readiness, and emergency response capacity contribute to avoidable deaths worldwide, establishing a clear conceptual foundation for equity-focused analyses of AED use. This framework aligns directly with this study’s examination of racial and geographic inequities in AED deployment during EMS encounters in the United States.

Out-of-hospital cardiac arrest (OHCA) remains a leading cause of mortality worldwide, with ventricular fibrillation accounting for a substantial proportion of sudden cardiac deaths [2,3,4]. Survival depends heavily on the interval between collapse and defibrillation, yet despite advances in EMS protocols, the average time to first shock often exceeds the critical five-minute window [5,6,7]. Public-access AEDs offer a practical means of reducing this delay by enabling lay responders and first-line professionals to deliver early defibrillation [8].

Extensive clinical and observational research demonstrates that early AED use significantly improves survival and neurological outcomes [7,9]. However, AED availability and use remain uneven across communities [10,11,12]. Home-use initiatives, rural regions, and under-resourced neighborhoods often experience lower AED access and reduced bystander intervention, raising concerns about equitable access to lifesaving interventions and the structural factors that shape prehospital care. This study addresses three equity-focused research questions:

1. Do racial disparities exist in AED use during EMS encounters?
2. Does AED use vary across geographic settings (urban, suburban, rural, frontier)?
3. Is AED application associated with the final patient acuity?

By integrating demographic, geographic, and clinical data from a large national EMS registry, this study provides new insight into how AED deployment patterns reflect broader inequities in prehospital care and identifies opportunities to strengthen AED access and readiness across diverse communities.

In this analysis, AED “use” reflects the NEMSIS variable eEquipment.24, which captures whether an AED was applied at any point during the EMS encounter. This definition does not distinguish between bystander AED use prior to EMS arrival and AED application initiated by EMS personnel. Because these two forms of AED use have different implications for public-access defibrillation policy, the findings presented here should be interpreted as reflecting overall AED deployment during EMS encounters rather than exclusively bystander-initiated defibrillation. This distinction is essential for contextualizing equity-focused analyses, as disparities in bystander AED use may differ from patterns observed in EMS-applied AEDs [13,14].

## 2. Literature Review

Automated external defibrillators (AEDs) were first introduced in the early 1980s to reduce the time between sudden cardiac arrest and defibrillation. While early devices were limited to clinical settings, modern AEDs have rapidly evolved into reliable, user-friendly tools that support widespread public-access defibrillation. Contemporary research increasingly emphasizes system-level deployment strategies and equity-focused approaches to improving AED availability and early defibrillation outcomes. Subsequent technological advancements, such as automated rhythm detection and simplified user interfaces, enabled safe use by lay responders and supported the expansion of public-access defibrillation programs [15,16].

Public-access defibrillation programs expanded rapidly in the 2000s, supported by legislative mandates and public health initiatives. AEDs became common in airports, shopping malls, schools, and office buildings, and consumer-oriented models made home acquisition feasible for high-risk individuals [17,18]. Modern AEDs incorporate self-testing batteries, wireless connectivity, and pediatric dose attenuators, improving reliability but increasing cost. Contemporary devices typically range from $1500 to $3000, with annual maintenance adding approximately $200 to $300 [19]. Despite these expenses, cost–benefit analyses consistently show that even modest survival gains justify AED program investments [19,20].

Public awareness and understanding of AEDs remain uneven. Surveys indicate that fewer than half of adults can locate the nearest AED in a crowded environment, and many confuse cardiac arrest with myocardial infarction—misconceptions that may delay intervention [21]. Although AEDs are designed for untrained users, formal training significantly increases confidence and willingness to act [11,22].

Early defibrillation within the first 3 to 5 min of witnessed ventricular fibrillation can raise survival rates from below 10% to as high as 70% [4,6]. Registry analyses confirm that bystander AED use improves survival to hospital discharge and favorable neurological outcomes by more than twofold. Pediatric protocols require reduced energy dosing, typically via attenuating pads or pediatric keys, to minimize myocardial injury [23,24].

Good Samaritan laws play a critical role in encouraging bystander AED use by reducing legal barriers to intervention. Federal and state statutes generally protect individuals who render emergency care in good faith, provided there is no gross negligence. Emerging evidence suggests that comprehensive liability protections correlate with higher rates of bystander AED application and shorter time to first shock [25,26].

Despite these advances, disparities persist. AED application is reported to be approximately three times more likely in majority White neighborhoods than in predominantly Black or Hispanic areas [27]. Rural and frontier communities experience AED use rates below 10%, compared to over 25% in urban centers [11]. These inequities persist even after adjusting for witnessed arrests and public location incidents [10,12].

EMS agencies with aggressive first-responder AED programs—equipping fire, police, and community volunteers—report bystander AED application rates exceeding 20%, compared to under 5% in areas relying solely on EMS personnel [28]. Legislative mandates require AEDs in many federal buildings, airports, and schools, but private sector placement varies widely [29,30].

Landmark investigations, including randomized trials and large registry analyses, consistently demonstrate that AEDs dramatically improve survival when deployed rapidly. Survival to hospital discharge doubled in communities equipped with AEDs and lay responder training programs [6]. Bystander AED use before EMS arrival has been associated with a nearly threefold increase in favorable neurological outcomes [31]. Programmatic factors such as device maintenance, responder organization, and ongoing evaluation also influence AED program success [17]. Even incremental increases in public-access to defibrillation can yield substantial public health gains [9].

The National Emergency Medical Services Information System (NEMSIS), established in 2001, standardizes EMS data collection across the United States. Its public release dataset provides aggregated access to more than 260 million EMS activations, enabling large-scale analyses of treatment patterns and outcomes [13,32,33]. The system’s uniform data elements support equity-focused research by allowing comparisons across demographic, geographic, and clinical subgroups.

## 3. Materials and Methods

This study used aggregated data from the National Emergency Medical Services Information System (NEMSIS) Public Release Data Cube, which provides summary counts of EMS encounters across the United States. The dataset includes incident-level information stratified by race and ethnicity, AED use (yes/no), geographic region (urban, suburban, rural, frontier), cardiac arrest status, and patient acuity. Because the dataset is aggregated, individual-level records were not available, and analyses were limited to summary-level patterns.

AED application was identified using variable eEquipment.24, which flags whether an AED was applied at any point during the EMS encounter. This measure does not distinguish between bystander-initiated AED use prior to EMS arrival and AED application performed by EMS personnel and therefore reflects overall AED deployment during the EMS response rather than true public-access AED use. Geographic classification was derived from eScene.21, which categorizes incident locations as urban, suburban, rural, or frontier based on NEMSIS definitions that incorporate population density, land-use characteristics, and federal rural–urban classification standards. Race and ethnicity were recorded under ePatient.35, and patient acuity was determined using standardized dispatch, provider impression, and disposition codes (eSituation.11–12; eDisposition.12). Together, these elements enabled an equity-focused analysis of AED deployment across demographic and geographic subgroups.

Descriptive statistics were used to calculate AED use rates and subgroup distributions. Because the dataset is aggregated and lacks individual-level covariates, no inferential statistical testing was conducted. All comparisons are descriptive and intended to illustrate broad patterns rather than statistical significance. Exploratory regression models were considered to examine broad associations; however, because the dataset is aggregated and lacks individual-level covariates, these models were not interpreted as inferential results and are therefore not presented. All findings are descriptive.

The use of the NEMSIS Public Release Data Cube was intentional and reflects the study’s descriptive epidemiologic focus. Individual-level NEMSIS data require agency participation agreements, data use approvals, and extensive data cleaning, which are beyond the scope of this exploratory equity analysis. The aggregated structure of the Data Cube provides standardized, nationally representative counts that allow for consistent comparisons across demographic and geographic subgroups. Because the dataset is not designed for inferential modeling or adjustment for confounding variables, all analyses in this study are descriptive and should be interpreted as patterns rather than evidence of inequity or causal relationships.

## 4. Results

The dataset includes 106,246 EMS encounters across six racial and ethnic groups. Of these encounters, 16,688 involved AED application (15.7%) and 89,558 did not (84.3%), providing a clear baseline for subgroup comparisons. Most incidents occurred in urban settings (86.0%), followed by rural (7.0%), suburban (4.9%), and frontier areas (1.4%). Cardiac arrest was documented in 12.9% of encounters. All results presented in this section are descriptive summaries of aggregated counts and are not based on inferential statistical testing.

AED use varied across racial groups. Asian patients experienced 1214 AED applications out of 5742 encounters (21.14%), American Indian or Alaska Native patients had 482 AED applications out of 2299 encounters (20.96%), and Black or African American patients had 4876 AED applications out of 23,319 encounters (20.91%). Hispanic or Latino patients had 3214 AED applications out of 17,528 encounters (18.34%), and Native Hawaiian or Other Pacific Islander patients had 184 AED applications out of 1001 encounters (18.39%). White patients had the lowest AED use rate, with 6718 AED applications out of 47,657 encounters (14.15%). These values are summarized in Table 1, which consolidates AED use counts, denominators, and rates across all racial groups for clarity and reproducibility.

Geographic patterns revealed additional disparities. Urban areas accounted for 14,355 AED applications out of 91,372 encounters (15.7%), while suburban areas had 412 AED applications out of 5207 encounters (7.9%). Rural regions had 1169 AED applications out of 7437 encounters (15.7%), and frontier areas had 152 AED applications out of 1230 encounters (12.4%). These figures indicate that AED deployment was similar in urban and rural settings, while suburban and frontier regions showed substantially lower use.

AED use was strongly associated with cardiac arrest status. Among the 13,712 encounters coded as cardiac arrest (12.9% of the dataset), 7214 involved AED application (52.6%). In contrast, among the 92,534 non-cardiac arrest encounters, only 9474 involved AED use (10.2%). These differences align with clinical expectations and confirm that AED deployment is concentrated in clinically severe encounters. Because the dataset provides only aggregated counts, measures of uncertainty such as confidence intervals or statistical significance cannot be calculated.

**Figure 1:**
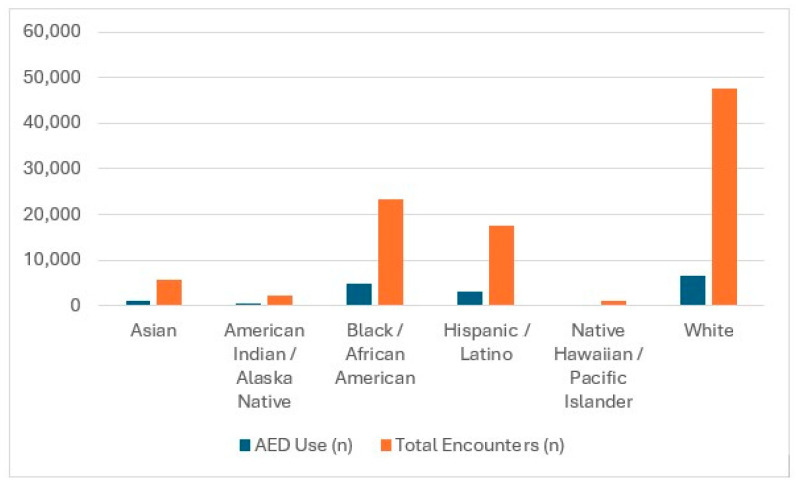
AED Use by Acuity Level and Race/Ethnicity.

Patient acuity further influenced AED deployment. AED use was most frequent among patients categorized as critical or deceased, reflecting the severity of these encounters. However, even within high-acuity categories, racial disparities persisted. For example, Black patients had a higher proportion of critical and death-coded encounters, yet their AED use rate was not proportionally elevated relative to other groups. These patterns are illustrated in Figure 1, which displays AED use across racial and ethnic groups. The figure highlights the descriptive differences observed in the dataset, with Asian, American Indian or Alaska Native, and Black or African American patients showing higher AED use rates than White patients, and Hispanic/Latino and Native Hawaiian/Pacific Islander patients falling in between. Taken together, these findings indicate that while AED use generally aligns with clinical indicators such as cardiac arrest and acuity, the observed racial and geographic differences represent descriptive patterns that warrant further investigation. Individual-level analyses are needed to determine whether these observed differences reflect incident characteristics, EMS system factors, or broader structural influences.

## 5. Discussion

This study contributes to the growing literature on AED effectiveness by examining racial and geographic inequities in AED deployment during EMS encounters. Because this study relied on aggregated NEMSIS Data Cube counts, the analyses are descriptive and cannot adjust for individual-level confounders such as comorbidities, socioeconomic status, or EMS agency characteristics. These limitations preclude causal inference and restrict the interpretation of observed associations. Rather than serving as inferential estimates, the findings highlight structural patterns that warrant deeper investigation using individual-level NEMSIS data or linked geospatial datasets. Consistent with prior research, AED use was closely tied to cardiac arrest status and clinical severity, underscoring the importance of early defibrillation for survival [7,9]. However, the analysis also revealed persistent disparities across racial and geographic groups, suggesting that AED access and EMS-associated deployment are not equitably distributed.

Despite these constraints, aggregated national EMS data provide a unique opportunity to identify large-scale demographic and geographic patterns in AED deployment. These descriptive disparities are important in their own right, as they reveal structural inequities in EMS-associated AED use that may not be visible in smaller or region-specific datasets.

Three key insights emerged. First, AED deployment varied substantially across racial groups. Although Asian, American Indian or Alaska Native, and Black or African American patients showed relatively high AED use rates, White patients—who accounted for the largest share of encounters—had the lowest rate. These patterns may reflect differences in incident characteristics, EMS protocols, or community-level AED availability, but they also raise concerns about structural inequities in prehospital care.

Second, geographic disparities were pronounced. Urban areas accounted for most AED deployments, whereas suburban and frontier regions showed markedly lower use, while rural AED use was similar to urban rates. These findings align with prior studies showing that AED access and EMS readiness are often concentrated in densely populated areas, leaving rural and frontier communities at a disadvantage [11]. Geographic inequities may stem from differences in AED placement, EMS response times, population density, and the availability of trained lay responders.

The geographic patterns observed in this study align with broader evidence demonstrating substantial regional variation in survival after cardiac arrest, independent of patient-level characteristics [6]. Prior research has shown that system-level factors, such as AED availability, emergency response organization, and bystander readiness, can profoundly influence outcomes.

Although this study highlights persistent inequities, it is also important to recognize that substantial progress has been achieved in some regions through coordinated public-access defibrillation strategies. A recent population-based analysis (Simmons et al., 2023) [34] demonstrated that comprehensive community engagement, strategic AED placement, and integrated emergency response planning can significantly increase bystander AED use and improve survival outcomes. These findings illustrate that disparities in early defibrillation are not immutable; targeted, system-level interventions can meaningfully strengthen community readiness and narrow geographic gaps in survival.

Geographic disparities in AED use may also reflect suboptimal placement strategies rather than simply a lack of devices. Evidence suggests that AED deployment optimized according to population density, movement patterns, and the spatial distribution of cardiac arrest events can substantially improve coverage and potential impact. A population-based modeling study (Chan et al., 2015) [35] demonstrated that aligning AED placement with real-world population flow—rather than relying solely on administrative or residential boundaries—significantly increased the proportion of cardiac arrests occurring within reach of an AED. This framework helps contextualize the present findings by highlighting that geographic inequities may stem from misaligned deployment strategies.

Third, even when AEDs were clinically indicated, such as in cardiac arrest or high-acuity encounters, racial disparities persisted. For example, Black patients had a higher proportion of critical and death-coded encounters, yet their AED use rate did not increase proportionally. This mismatch suggests that clinical severity alone does not fully explain AED deployment patterns and that broader systemic factors may influence prehospital treatment decisions.

Some of the observed patterns—such as higher AED use among certain racial and ethnic minority groups—should be interpreted with caution. These findings may reflect differences in incident mix, geographic concentration, or EMS deployment patterns rather than true equity in AED access. Observed racial differences may also arise from factors such as geographic distribution of populations, variation in incident types, and EMS system characteristics, rather than differential treatment or inequity in care delivery. For example, minority patients are disproportionately represented in densely populated urban areas where AED availability, bystander presence, and EMS readiness are typically higher. Similarly, variation in call types, incident locations, or agency-level protocols may influence AED deployment independent of patient characteristics. Accordingly, the observed racial and geographic differences should be understood as descriptive patterns rather than evidence of inequity, and interpretation should account for the contextual factors that cannot be measured in aggregated data.

It is also important to clarify that the AED use captured in this dataset reflects applications documented during EMS encounters and does not distinguish between bystander-initiated defibrillation and AED use performed by EMS personnel. As a result, the findings pertain to patterns of AED deployment within EMS response systems rather than true public-access or bystander AED use. This limitation may influence interpretation of racial and geographic differences, as EMS-driven AED use reflects agency protocols and response characteristics, whereas bystander AED use reflects community access, training, and device placement. Because these mechanisms cannot be separated in the dataset, the observed patterns should be interpreted cautiously. References to public-access defibrillation in this Discussion are intended to provide contextual grounding in the broader literature, not to imply that the present analysis directly measured pre-EMS AED application.

These findings have several practical implications. AED placement strategies should be aligned with spatial patterns of cardiac arrest incidence, prioritizing high-risk public and residential areas while expanding coverage in underserved communities. Standardized maintenance and training protocols can reduce variability in AED readiness and help ensure that devices function reliably when needed. Integrating AED location and status data into EMS dispatch systems may further reduce delays by guiding callers and responders to the nearest available device.

Community-based outreach and training initiatives are also essential. Cultural, linguistic, and socioeconomic barriers may affect willingness to use an AED or perform CPR, particularly in marginalized communities. Tailored education and engagement efforts can help address these barriers and promote equitable bystander intervention.

Future research should use individual-level data, quasi-experimental designs, and geospatial analyses to better identify causal pathways and guide targeted interventions. Qualitative studies involving community members, EMS personnel, and first responders may also illuminate behavioral and organizational factors that influence AED use before EMS arrival.

Overall, this study underscores the importance of integrating equity considerations into AED program design and implementation. By combining technological deployment with robust evaluation frameworks and inclusive community engagement, AED initiatives can more fully realize their potential to improve early response capacity and support equitable outcomes across diverse populations.

## 6. Limitations

This study has several limitations. Because the NEMSIS Public Release Data Cube provides only aggregated counts, individual-level analyses were not possible, and the models could not adjust for potential confounders such as comorbidities, socioeconomic status, or EMS agency characteristics. Additionally, key variables used in this analysis—including cardiac arrest status, patient acuity, and race and ethnicity—are derived from registry coding that may vary across EMS agencies, software systems, and regions. Differences in documentation practices, provider training, and local protocols can introduce heterogeneity in how these elements are recorded. As a result, some portion of the observed variation across demographic and clinical subgroups may reflect differences in coding practices rather than true differences in clinical presentation or AED deployment. This variability further reinforces the descriptive nature of the findings and the need for caution when interpreting subgroup differences.

Access to individual-level NEMSIS data requires agency-level participation agreements and data use approvals that were not feasible for this exploratory national-level analysis. Future work using individual-level NEMSIS data would allow for adjustment of patient and encounter-level covariates and support more robust inferential modeling. Additionally, the dataset does not include time-to-defibrillation metrics, which are central to understanding early intervention and survival outcomes and limit the ability to contextualize AED use within the broader chain of survival.

Aggregation also prevented assessment of within-group variability, clustering at the agency or county level, and interactions among demographic and clinical variables. Because analyses were conducted using aggregated counts, the study is subject to ecological fallacy, meaning that group-level patterns may not reflect individual-level relationships. The dataset does not include information on bystander characteristics, AED location, EMS response times, or the timing of AED application relative to collapse. These omissions limit the ability to determine whether disparities reflect differences in incident context, AED availability, EMS system performance, or broader structural factors. These constraints should be considered when interpreting the findings.

Finally, these findings reflect the structure of U.S. EMS systems, regulatory environments, and legal frameworks governing AED availability, Good Samaritan protections, and public-access defibrillation programs. Other countries differ substantially in EMS organization, dispatch models, responder training, and community AED infrastructure. As a result, the patterns observed in this study may not extrapolate directly to international settings, and caution is warranted when applying these results to healthcare systems with different operational or legal contexts. The descriptive patterns identified here should therefore be interpreted within the specific operational and regulatory landscape of the United States.

## 7. Conclusions

This descriptive national analysis identified racial and geographic differences in automated external defibrillator (AED) deployment during EMS encounters in the United States. Because the dataset captures any AED application documented during the EMS response without distinguishing between bystander-initiated use and EMS-applied devices, the findings reflect overall AED deployment patterns within EMS systems rather than disparities in public-access defibrillation specifically. AED use was strongly associated with cardiac arrest status and high patient acuity, yet descriptive differences persisted across racial and geographic groups, even within clinically severe encounters. Asian, American Indian or Alaska Native, and Black or African American patients exhibited higher AED use rates than White patients, and urban and rural settings showed similar AED use proportions, while suburban and frontier regions demonstrated substantially lower use. These patterns should be interpreted as group-level observations rather than evidence of inequity, as the aggregated dataset does not allow adjustment for confounding variables or assessment of causal pathways.

By focusing on aggregated national EMS data, the study highlights how demographic and geographic factors intersect with clinical severity to shape AED deployment patterns. The persistence of observed differences within high-acuity and cardiac arrest encounters is consistent with the possibility that system-level factors, such as AED placement, bystander readiness, and EMS deployment characteristics, may contribute to variation in AED use. However, the descriptive and ecological nature of the dataset prevents determining whether these patterns reflect inequity, incident context, or EMS system organization. Individual-level analyses are required to evaluate these mechanisms and to clarify whether the observed differences arise from structural, operational, or contextual factors.

Programmatic considerations remain important for strengthening early defibrillation capacity. Evidence from prior research underscores the value of strategic AED placement, standardized maintenance protocols, responder training, and integration of AED location data into EMS dispatch systems. Community-based outreach and training initiatives can help address cultural, linguistic, and socioeconomic barriers that influence willingness to intervene. These recommendations reflect established best practices in the broader AED and out-of-hospital cardiac arrest literature and are not causal inferences drawn from the descriptive patterns observed in this study.

Looking ahead, the expansion of public-access AED programs must emphasize both scale and equity. Future research using individual-level NEMSIS data, geospatial analyses, and linked EMS–hospital datasets is essential to identify the drivers of the descriptive patterns observed here and to evaluate how AED availability, community readiness, and EMS system characteristics influence early defibrillation. By coupling technological deployment with robust evaluation frameworks and inclusive community engagement, AED initiatives can strengthen early response capacity and help create the conditions that support improved survival outcomes across diverse populations.

## Figures and Tables

**Table 1:** Summary of AED Use Across Racial, Geographic, and Clinical Categories.

Category	Subgroup	AED Use (n)	Total Encounters (n)	AED Use Rate (%)
Race/Ethnicity	Asian	1214	5742	21.14
	American Indian/Alaska Native	482	2299	20.96
	Black/African American	4876	23,319	20.91
	Hispanic/Latino	3214	17,528	18.34
	Native Hawaiian/Pacific Islander	184	1001	18.39
	White	6718	47,657	14.15
Geographic Setting	Urban	14,355	91,372	15.7
	Suburban	412	5207	7.9
	Rural	1169	7437	15.7
	Frontier	152	1230	12.4
Cardiac Arrest Status	Cardiac Arrest	7214	13,712	52.6
	Not Cardiac Arrest	9474	92,534	10.2

## Data Availability

The data supporting the findings of this study are publicly available through the National Emergency Medical Services Information System (NEMSIS) Public Release Data Cube. This resource provides aggregated, de-identified EMS encounter data and can be accessed at: https://nemsis.org (accessed on 15 March 2026). No individual-level data were used or generated in this study.

## Abbreviations

The following abbreviations are used in this manuscript:

AED	Automated External Defibrillator
EMS	Emergency Medical Services
CPR	Cardiopulmonary Resuscitation
NEMSIS	National Emergency Medical Services Information System

## References

[1] Marijon E., Narayanan K., Smith K., Barra S., Basso C., Blom M.T., Crotti L., D’Avila A., Deo R., Dumas F. (2023). The Lancet Commission to reduce the global burden of sudden cardiac death: A call for multidisciplinary action. Lancet.

[2] Benjamin E.J., Muntner P., Alonso A., Bittencourt M.S., Callaway C.W., Carson A.P., Chamberlain A.M., Chang A.R., Cheng S., Das S.R. (2019). Heart disease and stroke statistics—2019 update: A report from the American Heart Association. Circulation.

[3] Harris P., Lysitsas D. (2016). Ventricular arrhythmias and sudden cardiac death. BJA Educ..

[4] Valenzuela T.D., Roe D.J., Nichol G., Clark L.L., Spaite D.W., Hardman R.G. (2000). Outcomes of rapid defibrillation by security officers after cardiac arrest in casinos. N. Engl. J. Med..

[5] Hansen C.M., Kragholm K., Granger C.B., Pearson D.A., Tyson C., Monk L., Corbett C., Nelson R.D., Dupre M.E., Fosbøl E.L. (2015). The role of bystanders, first responders, and emergency medical service providers in timely defibrillation and related outcomes after out-of-hospital cardiac arrest: Results from a statewide registry. Resuscitation.

[6] Hallstrom A.P., Ornato J.P., Weisfeldt M.L., Travers A.H., Christenson J., McBurnie M.A., Zalenski R., Becker L.B., Schron E.B., Proschan M. (2004). Public-access defibrillation and survival after out-of-hospital cardiac arrest. N. Engl. J. Med..

[7] Berdowski J., Blom M.T., Bardai A., Tan H.L., Tijssen J.G.P., Koster R.W. (2011). Impact of onsite or dispatched automated external defibrillator use on survival after out-of-hospital cardiac arrest. Circulation.

[8] Huebinger R., Blewer A.L. (2024). Public access defibrillation—Building toward a brighter future. JAMA Netw. Open.

[9] Pollack R.A., Brown S.P., Rea T., Aufderheide T., Barbic D., Buick J.E., Christenson J., Idris A.H., Jasti J., Kampp M. (2018). Impact of bystander automated external defibrillator use on survival and functional outcomes in shockable observed public cardiac arrests. Circulation.

[10] Garcia R.A., Spertus J.A., Girotra S. (2022). Racial and ethnic differences in bystander CPR for witnessed cardiac arrest. N. Engl. J. Med..

[11] American Heart Association (2013). Every Second Counts: Rural and Community Access to Emergency Devices. https://rescue-one.com/wp-content/uploads/aha-ruralcommunityaccess.pdf.

[12] Healio (2025). EMS Response to Cardiac Arrest Differs in Majority Black/Hispanic vs. White Catchment Areas. https://www.healio.com/news/cardiology/20250609/ems-response-to-cardiac-arrest-differs-in-majority-blackhispanic-vs-white-catchment-areas.

[13] Ehlers J., Fisher B., Peterson S., Dai M., Larkin A., Bradt L., Mann N.C. (2023). Description of the 2020 NEMSIS public-release research dataset. Prehospital Emerg. Care.

[14] NEMSIS Technical Assistance Center (2025). NEMSIS Version 3 Data Dictionary. https://nemsis.org/technical-resources/version-3/version-3-data-dictionaries/.

[15] American National Red Cross (2017). Responding to Emergencies: Comprehensive First Aid/CPR/AED.

[16] Ellingworth J. (2024). The History and Evolution of Defibrillation Technology. https://resources.defibshop.co.uk/blog/the-history-and-evolution-of-defibrillation-technology.

[17] Aufderheide T.P., Hazinski M.F., Nichol G., Steffens S.S., Buroker A., McCune R., Stapleton E., Nadkarni V., Potts J., Ramirez R.R. (2006). Community lay rescuer automated external defibrillation programs: Key state legislative components and implementation strategies. Circulation.

[18] Consumer Reports (2009). Should You Buy a Home Defibrillator?.

[19] Atlantic Health (2025). Automated External Defibrillator Program—Newton Medical Center Foundation. https://ahs.atlantichealth.org/about-us/foundations-auxiliaries/newton-foundation/aed-program.html.

[20] AED.ca (2024). The Cost-Benefit Analysis of Having an AED: Why Every Organization Should Consider It. https://aed.ca/blogs/news/the-cost-benefit-analysis-of-having-an-aed-why-every-organization-should-consider-it.

[21] Huang C.T., Chen C.H., Huang C.H. (2024). Public awareness of automated external defibrillator locations. JAMA Netw. Open.

[22] Liaw S.Y., Chew K.S., Zulkarnain A. (2020). Improving perception and confidence towards bystander CPR and AED use: How does training help?. Int. J. Emerg. Med..

[23] Samson R., Berg R., Bingham R. (2003). Use of automated external defibrillators for children: An update. Resuscitation.

[24] AED Brands Blog Can Adult AED Pads Be Used on Children? 2021. https://www.aedbrands.com/blog/can-you-use-adult-aed-pads-on-a-child/.

[25] Athea Trial Lawyers (2023). AED Lawsuits: Failure to Use Automated External Defibrillator. https://www.athealaw.com/practice-areas/premises-liability/aed-lawsuits/.

[26] Congress.gov (2023). H.R.1012—Cardiac Arrest Survival Act of 2023. https://www.congress.gov/bill/118th-congress/house-bill/1012/text.

[27] American Heart Association (2022). Black, Hispanic Adults Less Likely to Receive CPR, Especially in Public. https://www.heart.org/en/news/2022/05/13/black-hispanic-adults-less-likely-to-receive-cpr-especially-in-public.

[28] Folke F., Shahriari P., Hansen C.M., Gregers M.C.T. (2023). Public access defibrillation: Challenges and new solutions. Curr. Opin. Crit. Care.

[29] Centers for Disease Control and Prevention (2024). Automated External Defibrillators in Public Settings. https://www.cdc.gov/cardiovascular-resources/php/pad-slfs/index.html.

[30] CNN (2023). Is Your School Equipped to Save a Life?. CNN.

[31] Weisfeldt M.L., Sitlani C.M., Ornato J.P., Rea T., Aufderheide T.P., Davis D., Dreyer J., Hess E.P., Jui J., Maloney J. (2010). Survival after application of automatic external defibrillators before arrival of the emergency medical system: Evaluation in the Resuscitation Outcomes Consortium population of 21 million. J. Am. Coll. Cardiol..

[32] NEMSIS Technical Assistance Center (2025). History of NEMSIS. https://nemsis.org/what-is-nemsis/history-of-nemsis/.

[33] National Highway Traffic Safety Administration (2025). National Emergency Medical Services Information System (NEMSIS). https://www.transportation.gov/resources/individuals/privacy/national-emergency-medical-services-information-system-nemsis.

[34] Simmons K.M., McIsaac S.M., Ohle R. (2023). Impact of community based interventions on out of hospital cardiac arrest outcomes: A systematic review and meta analysis. Sci. Rep..

[35] Chan T.C.Y., Li H., Lebovic G., Tang S.K., Chan J.Y.C., Cheng H.C., Morrison L.J., Brooks S.C. (2015). Identifying locations for public access defibrillators using mathematical optimization. Circulation.

